# Nuclear cardiology in China: 2017

**DOI:** 10.1007/s12350-017-0985-x

**Published:** 2017-07-10

**Authors:** Gongshun Tang

**Affiliations:** West China Hospital, Sichuan University, 37 Guoxuexiang Street, Wuhou District, Chengdu, 610041 China

**Keywords:** SPECT, PET, MPI, nuclear cardiac imaging

## Abstract

This paper provides the current state of nuclear cardiology in China and contrasts it with the state of nuclear cardiology in the United States (US). The West China Hospital and New York-Presbyterian Hospital (NYPH) were used as representative hospitals to contrast nuclear cardiology in China and the US, respectively. In 2015, there were 101 medical cyclotrons, 774 SPECT or SPECT/CT, 240 PET/CT, and 6 PET/MR cameras in China. Most (~90%) of the nuclear cardiology studies are gated SPECT myocardial perfusion imaging (MPI), and ~10% are other types of studies including MUGA, PET/CT MPI, and viability studies. There are differences in nuclear cardiology between the West China Hospital and NYPH and these include those in cardiac stress tests, SPECT/CT acquisition protocols, PET/CT blood flow and viability studies, reimbursement, and fellowship training. In this paper, we aim to present status of nuclear cardiology in China and provide potential solutions.

## Introduction

The purpose of this article is twofold: (1) to provide information regarding the status of nuclear cardiology in China in 2016-2017, including current challenges and potential solutions, and (2) to present differences between nuclear cardiology in China and the US during this period. The first author spent one year at the New York-Presbyterian Hospital (NYPH)/Columbia University Medical Center (CUMC) nuclear cardiology laboratory, had previously worked in five hospitals in China during the past two decades, and has regularly participated in annual nuclear medicine meetings in China.

## Nuclear Cardiology Facility

Nuclear cardiology in China is not a separate division but is within the nuclear medicine division. There are a few hospitals in China designated for cardiac and vascular diseases but rarely do any of them have a nuclear cardiology laboratory. It was reported that there were 9467 nuclear medicine staff members in about 891 nuclear medicine laboratories in mainland China at the end of 2015, including 4012 (42.4%) nuclear medicine physicians, 2799 (29.6%) nuclear technologists, 1938 (20.5%) nurses, and 718 (7.6%) radiochemists, physicists, and engineers. In 2015 there were approximately 774 SPECT or SPECT/CT cameras, 240 PET/CT, 6 PET/MR, and 101 medical cyclotrons (Survey Report by Chinese Society of Nuclear Medicine in 2016, article in Chinese).^1^ Comparatively, in the US there were more than 2000 PET/CT cameras in 2016.

**Fig. 1:**
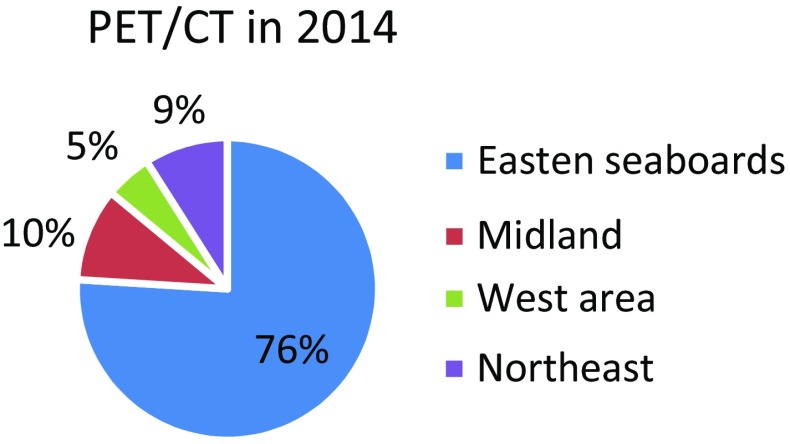
PET/CT distribution in China in 2014

Of note, there is an economic gap between the eastern seaboards and the mid-western regions of China. Almost 76% of the medical equipment for nuclear cardiology studies is located in the eastern seaboards and only ~15% in the mid-western regions of China (Figure 1).^2^ The majority of moderate-to-large hospitals (at least 1000 beds) have no PET/CT or even SPECT or SPECT/CT cameras in the mid-western regions of China. Of those hospitals with SPECT or SPECT/CT, a minority performed nuclear stress tests.

## Manpower and Training

In China, nuclear cardiology training is offered as a part of training in general nuclear medicine, with minimal amount of the curriculum focused on cardiac physiology, clinical cardiology, and radiology. As such, nuclear medicine training programs offer relatively less exposure to nuclear cardiology procedures. Consequently, there is a large knowledge gap amongst cardiologists and nuclear medicine radiologists performing nuclear cardiology procedures or referring patients to such procedures. In contrast to the US, before the year 2000, fellowship training was conducted in both teaching and non-teaching hospital in China, and it was only after 2000 that those training programs were restricted to teaching and approved large hospitals. The 5-year fellowship training programs of radiology and nuclear medicine in China do not require formal residency training. In contrast, the 5-year cardiology fellowship program in China requires formal residency training in internal medicine or in general surgery (Table 1).

**Table 1:** Medical education in US and China

	US	China
Admission	Completion of a first degree, “pre-med” courses, and medical college admission test	Graduated high school
Medical school	4 years	4 years
Rotating internship	Not required	1 year
Requirement for licensure to practice	3-step examination	2-step examination
Training required for nuclear cardiology	Formal nuclear medicine residency or cardiology fellowship, in a formal training program in a teaching hospital	Resident doctor in nuclear medicine department for 5 years in any hospital; does not need to be in a formal training program or in a teaching hospital. No training pathway for cardiologists
Training required for cardiology	Formal 3 year internal medicine residency and 3 + year cardiology fellowship, in formal training programs in a teaching hospital	Formal 5 year internal medicine residency and 5 year cardiology fellowship, in formal training programs in a teaching hospital
Board certification for nuclear medicine or nuclear cardiology	Yes	No
Board certification for cardiology	Yes	No

In total, as of 2014 there were 2.8 million physicians in China, including about 4000 nuclear medicine radiologists (~1/700). Of about 60,000 to 65,000 medical graduates in 2016, 100 to 200 of them selected to train in nuclear medicine.

## Nuclear Stress Tests

Both symptom limited exercise stress tests and 85% of maximal age-predicted heart rate (MPHR) stress tests are used in China.^3^ Most Chinese nuclear medicine physicians have limited expertise in cardiology, and as such 85% MPHR stress tests are perceived as safe to perform. In contrast, in the US at NYPH, symptom limited exercise stress tests are monitored by trained attending cardiologists. This is however not the case at all US laboratories; in many, exercise tests are monitored by mid-level practitioners or fellows.

## Pharmacological Stress Tests

Adenosine and regadenoson have not been approved by the China Food and Drug Administration (CFDA), and as such adenosine triphosphate (ATP) is used as an alternative pharmacologic stress agent.^4^ However, dipyridamole and dobutamine are approved by CFDA for pharmacological stress testing. The evidence base for ATP stress testing is not as robust as that for adenosine and regadenoson, and thus further research is needed regarding ATP stress testing.

## Stress/Rest Acquisition Protocols and Isotopes

Supine gated stress imaging, with either CT attenuation correction (CTAC) and/or prone non-gated imaging, is performed on all patients at NYPH. For most patients, stress imaging is performed first, with supine gated rest imaging performed only if warranted based on abnormal stress findings. For some patients with higher pre-test probability of abnormal findings, rest imaging is performed first. In China, either (i) supine gated stress imaging with CTAC, followed by supine gated rest imaging with CTAC, or (ii) supine gated stress imaging, followed by supine gated rest imaging, or (iii) prone non-gated stress imaging, followed by prone non-gated rest imaging, is performed using Tc-99m sestamibi for MPI tests. Although it has been approved by the CFDA, Tc-99m tetrofosmin is rarely used in China.

## Other Studies

Perfusion and viability studies using Tl-201, multi-gated acquisition (MUGA) studies for the assessment of left ventricular function, Tc-99m PYP studies for diagnosing cardiac amyloidosis, and adrenergic innervation studies using Iodine-123 metaiodobenzylguanidine (MIBG) are rarely performed in China.^5^,^6^ Measurement of myocardial blood flow using N-13 NH3 or Rb-82 with PET/CT is not regularly conducted in China.^7^ PET/CT F-18 FDG studies for myocardial viability and cardiac sarcoidosis are also rarely performed in China.^8^ In total, about 6 hospitals in China had a license for PET/MR in 2016.^9^-^13^

About 100,000 nuclear cardiology studies were performed in China in 2012, including about 3755 cardiac PET/CT studies (data published in Chinese). The annual test numbers did not change significantly from 2012 to 2016.

## Healthcare Insurance

In 2016, it was reported that China had a total population of over 1.4 billion inhabitants, with roughly 57% concentrated in urban areas. While China’s health care spending was estimated at 5.4% of the gross domestic product (GDP) in 2013, it was 17.4% of the US GDP in 2014.^14^ Tc-99m sestamibi MPI studies are not reimbursed for outpatients but only for inpatients. PET/CT scans are an out-of-pocket expense for most of the population.

Unlike in the US, patients with stable symptoms suspected to be due to CAD in China frequently undergo coronary angiography ($1000-1500) and coronary revascularization ($10,000 for CABG or for PCI) without prior functional assessment.

## Problems and Challenges

Nuclear cardiology in China has three main problems. First, high cost; for example, Tc-99m MIBI MPI costs $200-250 for a stress/rest study which few patients can afford. Second, turf issues exist between cardiology and nuclear medicine. This could be solved by fostering co-operative working relationships and establishment of nuclear cardiology laboratories run by either or both depending on qualifications. Third, there is a knowledge gap among physicians. This could be solved by a more structured training, exchange programs and additional training outside China, and visiting lectureships by experts in the field.

## Conclusion

In China, nuclear cardiology is a young field. Further development is required and obstacles need to be overcome before nuclear cardiology becomes widely available for routine patient care, to advance cardiovascular health across the population.

## Abbreviations

PET: Positron emission tomography
SPECT: Single photon emission computerized tomography
Tc-99m MIBI: Technetium-99m sestamibi
N-13 NH3: N-13 ammonia
FDG: Fluorodeoxyglucose
Tl-201: Thallium-201
ATP: Adenosine triphosphate
CAD: Coronary artery disease
MPHR: Maximally predicted heart rate
